# Draft genome sequence of *Fusobacterium necrophorum* subsp. *necrophorum* FNN105 isolated from the oral cavity of an alpaca (*Vicugna pacos*)

**DOI:** 10.1128/mra.00530-26

**Published:** 2026-10-15

**Authors:** Justine Kilama, Devin B. Holman, Raghavendra G. Amachawadi, T. G. Nagaraja, Carl R. Dahlen, Samat Amat

**Affiliations:** 1 Department of Plant Pathology, Microbiology and Biotechnology, North Dakota State University 3323, Fargo, North Dakota, USA; 2 Department of Animal Sciences, Center for Nutrition and Pregnancy, North Dakota State University 3323, Fargo, North Dakota, USA; 3 Lacombe Research and Development Centre, Agriculture and Agri-Food Canada 98668, Lacombe, Alberta, Canada; 4 Department of Clinical Sciences, College of Veterinary Medicine, Kansas State University 5308, Manhattan, Kansas, USA; 5 Department of Diagnostic Medicine and Pathobiology, College of Veterinary Medicine, Kansas State University 5308, Manhattan, Kansas, USA; Nanchang University, Nanchang, Jiangxi, China

**Keywords:** *Fusobacterium necrophorum*, alpaca, draft genome sequence, whole-genome sequencing, camelid

## Abstract

*Fusobacterium necrophorum* is frequently associated with oral cavity infections in livestock and other animal species. Here, we present the draft genome sequence of *F. necrophorum* subsp. *necrophorum* FNN105, isolated from the oral cavity of an alpaca (*Vicugna pacos*). The draft genome is 2.49 Mbp and contains 2,237 predicted protein-coding sequences.

## ANNOUNCEMENT

*Fusobacterium necrophorum* is a gram-negative, anaerobic bacterium associated with a variety of necrotic infections in animals and humans. In cattle, it is a major pathogenic agent involved in liver abscesses, necrotic laryngitis, and foot rot, while in deer and other wild animals, it has been associated with laryngitis, periodontal infections, and mandibular abscesses. In humans, it is linked to sore throat and Lemierre’s syndrome (1–4). Although *F. necrophorum* has been detected in various host-associated niches, genomic data from camelids remain limited (5, 6). Here, we present the draft genome sequence of *F. necrophorum* subsp. *necrophorum* isolate FNN105, isolated from the oral cavity of an alpaca (*Vicugna pacos*). An oral cavity sample collected from an alpaca with suspected laryngeal infection was submitted as a clinical case and processed for the anaerobic isolation of *Fusobacterium* as previously described (7). The sample was enriched in pre-reduced, anaerobically sterilized peptone-yeast extract medium supplemented with 100 mM lactate and lysine, plus josamycin (3 µg/mL), vancomycin (4 µg/mL), and norfloxacin (1 µg/mL). Enrichment cultures were incubated anaerobically at 37°C for 24 h, streaked onto blood agar plates, and incubated anaerobically at 37°C for 48 h (7, 8). Presumptive *Fusobacterium* colonies were selected based on colony morphology and subcultured in brain heart infusion broth (8). Species and subspecies identification was confirmed by qPCR targeting the promoter of the leukotoxin gene (*lktA-n*) using forward primer 5′-GCTTTGGAAGAAGCCAAACA-3′, reverse primer 5′-AATGCTTCCATTCGGATTCA-3′, and probe 5′-FAM-TGGAATCATTCCAGTAGATGGAAAAG-ZEN/3′IB-3′ (9). Isolate FNN105 was identified as *F. necrophorum* subsp. *necrophorum*.

Genomic DNA was extracted from an overnight culture using the DNeasy Blood and Tissue Kit. Whole-genome sequencing was performed using the NovaSeq X Plus platform with paired-end 150 bp sequencing, generating 14,952,090 read pairs. Libraries were prepared using the VAHTS Universal DNA Library Prep Kit for Illumina V4. Raw reads were adapter-trimmed and quality-filtered using fastp v0.23.2 (10). *De novo* assembly was performed with SPAdes v4.2.0 using the “--isolate” flag (11). Assembly quality was evaluated using QUAST v5.2.0 (12), and genome completeness and contamination were assessed using CheckM2 v1.1.0 (13). Genome annotation was performed using the NCBI Prokaryotic Genome Annotation Pipeline (PGAP v6.11). Virulence-associated genes (VF) were identified using DIAMOND BLASTP v2.1.15 (14) against a combined database comprising the VF Database (15) and a curated *F. necrophorum*–specific virulence database (16), using thresholds of ≥80% amino acid identity and ≥80% query coverage. Antimicrobial resistance genes (ARGs) were screened using Resistance Gene Identifier v6.0.3 with CARD v4.0.1 (17). Default parameters were used except where otherwise noted.

The draft genome assembly comprised 101 contigs totaling 2,486,085 bp, with an *N*_50_ of 53,967 bp, genome coverage of 192.3×, and a maximum contig length of 136,100 bp. Genome completeness and contamination were 100% and 0.59%, respectively. The genome had a G + C content of 34.03% and a coding density of 90.5%. There were 2,237 predicted protein-coding sequences, 8 rRNA genes, 42 tRNA genes, 3 ncRNAs, 48 pseudogenes, and 7 CRISPR arrays. Virulence-associated genes included leukotoxin genes (*lktA*, *lktB*, and *lktC),* adhesins, autotransporter proteins, and immune evasion-associated factors. No ARGs were detected.

## Data Availability

This Whole Genome Shotgun project has been deposited in DDBJ/ENA/GenBank under accession number JBYBGY000000000. The version described in this paper is the first version, JBYBGY000000000.1. Raw sequencing reads are available in the Sequence Read Archive under BioProject PRJNA1446192, BioSample SAMN56814162, and accession number SRR37881993.

## References

[1] Nagaraja TG, Chengappa MM. 1998. Liver abscesses in feedlot cattle: a review. J Anim Sci 76:287–298. doi:10.2527/1998.761287x 9464910

[2] Riordan T. 2007. Human infection with *Fusobacterium necrophorum* (*Necrobacillosis*), with a focus on lemierre’s syndrome. Clin Microbiol Rev 20:622–659. doi:10.1128/CMR.00011-07 PMC2176048 17934077

[3] Antiabong JF, Boardman W, Smith I, Brown MH, Ball AS, Goodman AE. 2013. A molecular survey of a captive wallaby population for periodontopathogens and the co-incidence of *Fusobacterium necrophorum* subspecies necrophorum with periodontal diseases. Vet Microbiol 163:335–343. doi:10.1016/j.vetmic.2013.01.012 23428381

[4] Roeder BL, Chengappa MM, Lechtenberg KF, Nagaraja TG, Varga GA. 1989. *Fusobacterium necrophorum* and actinomyces pyogenes associated facial and mandibular abscesses in blue duiker. J Wildl Dis 25:370–377. doi:10.7589/0090-3558-25.3.370 2761010

[5] Kumar A, Anderson D, Amachawadi RG, Nagaraja TG, Narayanan SK. 2013. Characterization of *Fusobacterium necrophorum* isolated from llama and alpaca. J Vet Diagn Invest 25:502–507. doi:10.1177/1040638713491407 23780933

[6] Carvallo FR, Uzal FA, Flores C, Diab SS, Giannitti F, Crossley B, Wünschmann A. 2020. Alimentary necrobacillosis in alpacas. J Vet Diagn Invest 32:339–343. doi:10.1177/1040638720906409 PMC7081513 32070228

[7] Schwarz C, Mathieu J, Gomez JL, Miller MR, Tikhonova M, Nagaraja TG, Alvarez PJJ. 2023. Unexpected finding of *Fusobacterium varium* as the dominant *Fusobacterium species* in cattle rumen: potential implications for liver abscess etiology and interventions. J Anim Sci 101:skad130. doi:10.1093/jas/skad130 PMC10195197 37104065

[8] Kilama J, Dahlen CR, Abbasi M, Shi X, Nagaraja TG, Crouse MS, Cushman RA, Snider AP, McCarthy KL, Caton JS, Amat S. 2025. Characterizing the prevalence of *Fusobacterium necrophorum* subsp. *necrophorum, Fusobacterium necrophorum* subsp. *funduliforme,* and *Fusobacterium varium* in bovine and ovine semen, bovine gut, and vagino-uterine and fetal microbiota using targeted culturing and qPCR. Microbiol Spectr 13:e0314524. doi:10.1128/spectrum.03145-24 PMC12054004 40126218

[9] Deters A, Shi X, Bai J, Kang Q, Mathieu J, Nagaraja TG. 2024. A real-time PCR assay for the detection and quantification of *Fusobacterium necrophorum* and *Fusobacterium varium* in ruminal contents of cattle*. Applied Animal Science 40:250–259. doi:10.15232/aas.2023-02507

[10] Chen S. 2025. Fastp 1.0: an ultra-fast all-round tool for FASTQ data quality control and preprocessing. Imeta 4:e70078. doi:10.1002/imt2.70078 PMC12527978 41112039

[11] Prjibelski A, Antipov D, Meleshko D, Lapidus A, Korobeynikov A. 2020. Using SPAdes *de novo* assembler. Curr Protoc Bioinformatics 70:e102. doi:10.1002/cpbi.102 32559359

[12] Gurevich A, Saveliev V, Vyahhi N, Tesler G. 2013. QUAST: quality assessment tool for genome assemblies. Bioinformatics 29:1072–1075. doi:10.1093/bioinformatics/btt086 PMC3624806 23422339

[13] Chklovski A, Parks DH, Woodcroft BJ, Tyson GW. 2024. CheckM2: a rapid, scalable and accurate tool for assessing microbial genome quality using machine learning. Nat Methods 21:735. doi:10.1038/s41592-024-02248-z 38514780

[14] Buchfink B, Reuter K, Drost HG. 2021. Sensitive protein alignments at tree-of-life scale using DIAMOND. Nat Methods 18:366–368. doi:10.1038/s41592-021-01101-x PMC8026399 33828273

[15] Chen L, Yang J, Yu J, Yao Z, Sun L, Shen Y, Jin Q. 2005. VFDB: a reference database for bacterial virulence factors. Nucleic Acids Res 33:D325–8. doi:10.1093/nar/gki008 PMC539962 15608208

[16] Carrara A, Bertelli C, Gardiol C, Marquis B, Andrey DO, Schrenzel J, Pillonel T, Greub G. 2024. Association of pathogenic determinants of *Fusobacterium necrophorum* with bacteremia, and Lemierre’s syndrome. Sci Rep 14:19804. doi:10.1038/s41598-024-70608-y PMC11350119 39191804

[17] Alcock BP, Huynh W, Chalil R, Smith KW, Raphenya AR, Wlodarski MA, Edalatmand A, Petkau A, Syed SA, Tsang KK, et al.. 2023. CARD 2023: expanded curation, support for machine learning, and resistome prediction at the comprehensive antibiotic resistance database. Nucleic Acids Res 51:D690–D699. doi:10.1093/nar/gkac920 PMC9825576 36263822

